# Clinical presentation of acute appendicitis in Babol; northern of Iran

**DOI:** 10.22088/cjim.8.2.129

**Published:** 2017

**Authors:** Kamal Rashidi-Azar, Ali-Asghar Darzi, Sekineh Kamali-Ahangar, Sepideh Siadati, Abdolrahim Gholizadehpasha

**Affiliations:** 1 Department of General surgery, Shahid Beheshti Hospital, Babol University of Medical Sciences, Babol, Iran.; 2 Cancer Research Center, Health research Institute, Babol University of Medical Sciences, Babol, Iran.; 3 Clinical Research Development Center, Shahid Beheshti Hospital, Babol University of Medical Sciences, Babol, Iran.; 4 Department of Pathology, Babol University of Medical Science, Babol, Iran.

**Dear Editor,**

Acute appendicitis is the most common cause of abdominal pain that leads to surgery. About 10 % of people for are affected by this disease throughout life (1). More than half of patients with appendicitis had history of vague abdominal pain, nausea and anorexia. Finally, pain is shifted to the right lower quadrant of abdomen (2, 3). It should be noted that the prevalence of age, gender and symptoms have been different in different regions (4). Any delay in diagnosis lead to complications with increased morbidity and mortality (5). Because of the lack of study in this field in northern Iran, we decided to investigate the epidemiology of acute appendicitis in this region.

A total of 219 patients (151 males, 68 females) with acute appendicitis admitted to Shahid Beheshti Hospital, Babol, No‌rthern Iran during 2014 to 2015 were enrolled. The mean age was 31.35±13.63 years (range 11-84). Most of the patients were 21-30 years old(35.6%) that was similar to the study by Davood Abadi. et .al (6). However, some studies reported that the most common cases occurred in the second decade of life (7).

In our study, male to female ratio was 2.3 to 1 which was different from some studies (8). Pain shift (n=174, 79.5%) was the most common symptom followed by anorexia, that was different from Fallon et. al’s who mentioned that anorexia was seen in more than 95% of patients (9). This difference may be influenced by different diets. In the current study, leukocytosis was seen in 81.7% of patients. In literature, there are many controversial studies about leukocytosis. In our study, periumbilical area with 72 (32.9%) cases was the most common site of pain and hypogasteric with 4 (1.8%) cases was the least site.

On physical examination, tenderness and rebound tenderness were seen in 45.7 % of patients in RLQ and tenderness and rebound tenderness guarding were seen in 43.4 % of patients. Totally, 218 (99.5 %) patients had tenderness in RLQ, 200 (91.3%) cases with rebound tenderness in RLQ and 101 (46.1%) patients had guarding. In this study, 82.3% of patients with appendicitis had alvarado score more than 7. Nevertheless, among patients with no appendicitis in pathological examination, 53.6% of then had the same score. Up to now; tissue diagnosis remains the gold standard treatment acute of appendicitis. For more accurate preoperative diagnosis, physical examination, ultrasonography and laboratory investigation have been used. 

With history taking and physical examination, the diagnosis was reached in 80% of patients. With the help of ultrasonography diagnosis could be increased to 98%; nonetheless, there is still disagreement about the role of this method in accurate diagnosis of appendicitis. In the current study based on pathological examination 171 cases had appendicitis and 31 did not. Suppurative appendicitis was the most common type seen on pathological examination that was different from Kiselev et.al.’s study (10).

In conclusion, the clinical presentation in our study was different from other studies. Anorexia was not the most common symptom (77.6%) and age range 21 to 30 was the most common age of acute appendicitis. The ratio of male to female in this study was higher than other studies.
